# Influence of Anisotropic White Matter on Electroosmotic Flow Induced by Direct Current

**DOI:** 10.3389/fbioe.2021.689020

**Published:** 2021-08-13

**Authors:** Teng Wang, Svein Kleiven, Xiaogai Li

**Affiliations:** Division of Neuronic Engineering, Department of Biomedical Engineering and Health Systems, KTH Royal Institute of Technology, Huddinge, Sweden

**Keywords:** cerebral edema, anisotropic conductivity, electroosmotic flow, electroosmosis based approach, finite element head model

## Abstract

Treatment of cerebral edema remains a major challenge in clinical practice and new innovative therapies are needed. This study presents a novel approach for mitigating cerebral edema by inducing bulk fluid transport utilizing the brain’s electroosmotic property using an anatomically detailed finite element head model incorporating anisotropy in the white matter (WM). Three representative anisotropic conductivity algorithms are employed for the WM and compared with isotropic WM. The key results are (1) the electroosmotic flow (EOF) is driven from the edema region to the subarachnoid space under an applied electric field with its magnitude linearly correlated to the electric field and direction following current flow pathways; (2) the extent of EOF distribution variation correlates highly with the degree of the anisotropic ratio of the WM regions; (3) the directions of the induced EOF in the anisotropic models deviate from its isotropically defined pathways and tend to move along the principal fiber direction. The results suggest WM anisotropy should be incorporated in head models for more reliable EOF evaluations for cerebral edema mitigation and demonstrate the promise of the electroosmosis based approach to be developed as a new therapy for edema treatment as evaluated with enhanced head models incorporating WM anisotropy.

## Introduction

Cerebral edema is a common clinical problem defined as an abnormal accumulation of excess fluid in the brain’s extracellular or intracellular space, which is a significant cause of mortality (Donkin and Vink, 2010; Walcott et al., 2012). The development of cerebral edema is a complex physiologic and pathologic process associated with tumor, hemorrhage, stroke, traumatic brain injury (TBI), and infection (Jha et al., 2019). Especially cerebral edema caused by TBI is often associated with raised intracranial pressure (ICP) and risks of irreversible brain injury, herniation, and death unless treated effectively (Jha et al., 2019). The primary goals for treating edema with increased ICP are to regulate cerebral perfusion and reduce the ICP. Most existing treatments are non-specific and target towards ameliorating the effect of edema, such as hyperosmolar treatment, neuromuscular blockade, hypothermia, sedation, and decompressive craniectomy (DC) (Walcott et al., 2012; Jha et al., 2019; Hale et al., 2020); most options are accompanied with significant side effects (Li et al., 2013; Jha et al., 2019). Thus, the treatment of cerebral edema is still an arduous task, and new treatment approaches are to be sought.

The electroosmotic property of brain tissue is relatively well understood (Guy et al., 2009; Cancel et al., 2018; Faraji et al., 2019) and has been applied in various applications (Rupert et al., 2013; Faraji et al., 2019; Faraji et al., 2020). However, the application for edema treatment has not been explored until recently (Wang et al., 2020). In a previous study, we proposed a novel electroosmosis based approach for cerebral edema treatment by applying a direct current to the head (Wang et al., 2020). Using an anatomically detailed head model, we investigated the feasibility and safety of the approach, showing its promise as a potential treatment for driving edematous tissue fluid by applying direct current. The mechanism is to utilize brain tissue’s electroosmotic property as the brain tissue can be regarded as a conductive and electrically active matrix, filled with extracellular fluid as a strong electrolyte due to the existence of dissociated ionic compounds (Savtchenko et al., 2017). The negatively charged surface of the phospholipid cell membrane attracts a large number of cations dissociating in the extracellular fluid space, forming an electrical double layer (EDL) (Helmholtz, 1879). When an external electric field is applied on an electrolyte-filled porous matrix such as the brain, the movement of cations driven by electric forces pull along the adjacent water molecules through a friction function, resulting in bulk fluid flow, namely electroosmotic flow (EOF) (Ou et al., 2014; Savtchenko et al., 2017).

White matter (WM) has been shown to have anisotropic electrical conductivity (Lee et al., 2012; Abderezaei et al., 2019). WM fiber architecture plays a key role in the electric field distribution and current flow pathways within the brain. Several different algorithms have been proposed to estimate the WM anisotropic conductivity from diffusion tensor imaging (DTI), which has been used to simulate electric field in head models (Hallez et al., 2008; Shahid et al., 2013; Huang et al., 2017). Previous head models with anisotropic WM showed significantly different current density distribution in transcranial direct current stimulation (tDCS) or source localization in electroencephalography than models with isotropic WM (Hallez et al., 2008; Shahid et al., 2013). In contrast, Huang et al. (2017) compared the electric field distribution on the brain surface in isotropic and anisotropic models and concluded that the anisotropic WM did not improve the prediction accuracy. The inconsistent results appear to suggest that whether to incorporate anisotropic WM depends on specific applications. Especially when applied for edema treatment evaluation, isotropic conductivity for WM has been used in our previous study (Wang et al., 2020), and it’s yet to be explored how WM anisotropic conductivity may improve the reliability of EOF prediction, as EOF value is proportional to the electric field magnitude and its direction is parallel to the electric current flow pathway.

Finite element (FE) method allows handling complex geometries and boundary conditions, and FE head models have emerged as powerful numerical tools to solve partial differential equations in many fields within neuroscience (Huang et al., 2017; Anderson et al., 2020; Li et al., 2020; Zhou et al., 2020). In this study, an anatomically detailed FE head model is employed to investigate the influence of WM anisotropy on the treatment efficiency of our previously proposed electroosmosis based approach by studying the induced EOF. For this purpose, three anisotropy algorithms are implemented for the WM, and validation for all models is performed to verify the accuracy of model predictions compared to measured values from patients reported earlier (Huang et al., 2017).

## Materials and Methods

### Theory of Electroosmosis

Electroosmosis is a fundamental electrokinetic phenomenon first observed by Reuss (1809) in 1808 involving movement of the bulk solution against a charged solid surface under the influence of an electric field defined as EOF towards the cathode, which can be quantified by Helmholtz–Smoluchowski approximation Helmholtz (1879). EOF is possible because of the presence of EDL at the porous interface with non-zero zeta-potential (Wiley and Weihs, 2016). In the case of brain tissue, previous experimental studies have measured zeta-potential of brain tissue (Guy et al., 2008; Guy et al., 2009), which confirmed brain tissue’s electroosmotic property. Moreover, the Helmholtz–Smoluchowski approximation has also been incorporated in calculating the electroosmotic perfusion of brain tissues to investigate the fluid flow inside the brain (Ou et al., 2014; Ou and Weber, 2017; Faraji et al., 2020). Based on above, we hypothesize that edematous fluid could be driven out of the brain by an applied direct current to alleviate brain edema.

### Electroosmotic Flow Modelling

When an external electric current is applied to the brain, the cations in the extracellular space move along the narrow channels under the action of the electric field. Then the adjacent water is directed to flow with the cations due to the viscous drag (Guy et al., 2008; Guy et al., 2009; Savtchenko et al., 2017), generating EOF along the narrow channels.

The velocity of the induced EOF flow across the brain is governed by Helmholtz–Smoluchowski approximation as follows:

$$\text{ν}=-\frac{\varepsilon_{r}\varepsilon_{0}\zeta E}{\eta }$$ (1)

where ν represents the EOF velocity, $\varepsilon_{r}$ is the relative permittivity of the extracellular solution (84.6), $\varepsilon_{0}$ is the vacuum permittivity (8.85 × 10^–12^ F/m), *E* is the electric field, and ζ represents the zeta-potential of brain tissue (−22.8 mV). These values are taken from the literature related to the zeta-potential measurement of rat brains (Guy et al., 2008; Guy et al., 2009). Given that the direct current stimulation enlarges the extracellular space and increases the effective solute diffusion coefficient of brain tissue (Avramov, 2009; Xia et al., 2020), the viscosity η of the extracellular solution is adjusted to be 5.8 × 10^–4^ Pas.

### Electric Field Modelling

As seen from Eq. 1, to calculate EOF velocity inside the brain, a distribution of the electrical field in the brain is a prerequisite. The distribution of the electric field is governed by Laplace’s equation under quasi-stationary conditions:

∇⋅(−σ∇V)=0 (2)

where ∇ denotes the gradient vector,  V is the electric potential, and σ is the electrical conductivity of the tissue. The electric field, *E*, is derived from the electric potential as:

E=−∇V (3)

The current density J of volume conductor is calculated according to Ohm’s law:

J=σE (4)

### Electric Conductivity and Anisotropic WM Modelling

The conductivity values are set to 0.435 S/m for scalp, 0.029 S/m for cancellous bone, 0.01 S/m for cortical bone, 0.53 S/m for dura mater, 1.79 S/m for CSF, 0.333 S/m for GM, 1.79 S/m for ventricular system, and 0.1428 S/m for isotropic WM (Gabriel, 1996; Haueisen et al., 1997; Ramon et al., 2006; Wang et al., 2020). The conductivity values of the electrode and sponge are assigned as 5.99 × 10^7^ S/m and 1.4 S/m, respectively (Truong et al., 2013). For the WM, three representative anisotropic conductivity algorithms are implemented including, the proportional anisotropic ratio (PRO) algorithm, equivalent isotropic trace (EQU) algorithm, and fixed anisotropic ratio (FIX) algorithm, with details provided below. Besides, an isotropic model (ISO) is also implemented for WM for comparison.

#### Proportional Anisotropic Ratio Algorithm

The PRO algorithm is suggested by Hallez et al. (2008) based on effective medium approach. The eigenvalues of the conductivity tensors σ at a voxel level are calculated based on a linear relationship between the eigenvalues of the diffusion tensor and the conductivity tensor defined in Eq. 5:

$$\frac{d_{1}}{\sigma_{1}}=\frac{d_{2}}{\sigma_{2}}=\frac{d_{3}}{\sigma_{3}}$$ (5)

where $d_{1}$, $d_{2}$, and $d_{3}$ denote the eigenvalues of the diffusion tensor at each WM voxel, and $\sigma_{1}$, $\sigma_{2}$, and $\sigma_{3}$ are the eigenvalues of conductivity tensor at corresponding voxel. Then the volume constraint is incorporated to calculate the eigenvalues of the conductivity tensor based on the theory of keeping the volume of the isotropic tensor and anisotropic tensor same (Wolters et al., 2006), i.e.

$$\frac{4}{3}\pi \sigma_{iso}^{3}=\frac{4}{3}\pi \sigma_{1}\sigma_{2}\sigma_{3}$$ (6)

where $\sigma_{iso}$ represents the WM isotropic conductivity with a value of 0.1428 S/m.

#### Equivalent Isotropic Trace Algorithm

The EQU algorithm is proposed by Miranda et al. (2001). The eigenvalues of conductivity tensor are calculated by multiplying the diffusion tensor eigenvalue at each voxel by the ratio of the isotropic conductivity trace (3$\sigma_{iso}$) to the diffusion tensor trace according to Eq. 7:

$$\sigma_{i}=\frac{3\sigma_{iso}}{trace\left(D\right)}d_{i}$$ (7)

where D denotes the diffusion tensor, $d_{i}$ denote the eigenvalues of diffusion tensor at each WM voxel, and $\sigma_{i}$ are the eigenvalues of conductivity tensor at corresponding voxel (i = 1, 2, and 3).

#### Fixed Anisotropic Ratio Algorithm

The FIX algorithm uses a fixed anisotropic ratio among transverse and longitudinal conductivity. The anisotropic conductivity ratio of 1:9 between transverse and longitudinal conductivity is adopted to calculate the WM anisotropic conductivity (Hallez et al., 2008), i.e.

$$\sigma_{1}=9\cdot \sigma_{2}\;,\;\sigma_{2}=\sigma_{3}$$ (8)

where $\sigma_{1}$ is the largest eigenvalue along the longitudinal eigenvector of the diffusion tensor, $\sigma_{2}$ and $\sigma_{3}$ are the eigenvalues along the transverse eigenvector. Then the volume constraint equation (Eq. 6) is incorporated to calculate the eigenvalues of conductivity tensor.

### Finite Element Simulation of EOF

#### F E Head Model Development

**FIGURE 1:**
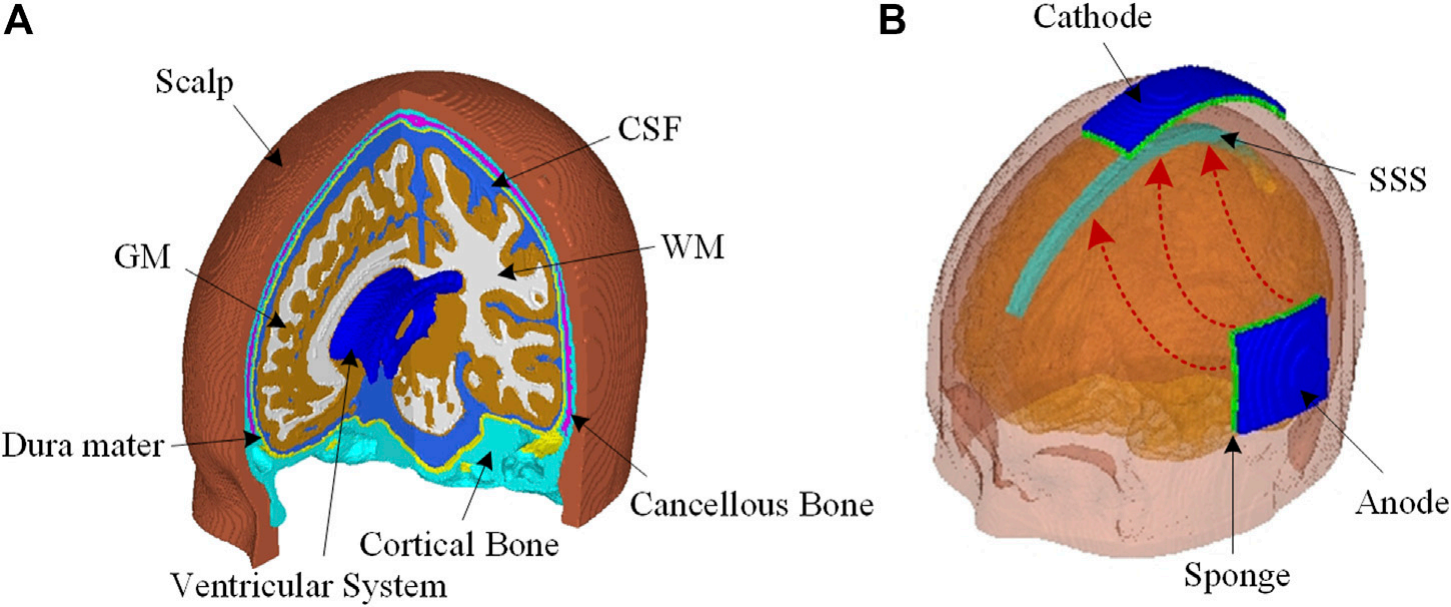
**(A)** The FE head model consists of the scalp, cortical bone, cancellous bone, dura mater, CSF, GM, WM, and a complete ventricular system. **(B)** Head model configuration for EOF treatment with an anode placed at the left side of the head and a cathode at the top of the head. The excess fluid is expected to be driven out of edema region underneath the anode to flow into SAS, and then absorbed into SSS together with CSF.

The ICBM 152 atlas, including T1W, T2W, and probability maps (Fonov et al., 2011), is used to develop a realistic FE head model. The MR images, together with the spatial information provided by the probability maps, are segmented into eight components using the Expectation-Maximization (EM) algorithm implemented in the open-source software 3D Slicer (Pieper et al., 2004). Hexahedral elements are then generated utilizing an in-house code based on a smoothed-voxel algorithm presented by Boyd et al. (2006). The resultant mesh consists of approximately 3.45 million hexahedral elements with a mesh resolution of 1 mm. The FE head model contains eight different sub-regions, including the scalp, cancellous bone, cortical bone, dura mater, cerebrospinal fluid (CSF), gray matter (GM), WM, and ventricular system (Figure 1A).

For electrode configuration, the anode pad (5 × 5 cm) is close to the brain area where extra tissue fluid is intended to be drawn out; the cathode pad (3 × 10 cm) is located at the top of the head above the subarachnoid space (SAS) to facilitate the extra fluid to be absorbed into superior sagittal sinus (SSS) together with CSF (Figure 1B). The outer surface of the anode is set as 15 V, and the cathode is set as 0 V. All other external surfaces of the head model are assigned to be electrically insulated. The value of 15 V at the anode is chosen *via* a trial-and-error approach to keep the induced current density and temperature within safety level.

#### Incorporating DTI for WM Anisotropic Conductivity

The diffusion tensor, including eigenvalues and eigenvectors at each WM voxel, is calculated from the ICBM DTI-81 atlas (Mori et al., 2008). For the three representative anisotropic conductivity algorithms, the eigenvalues of the conductivity tensors at each WM voxel are calculated, and the conductivity tensors share eigenvectors with the diffusion tensors. Given the geometry of the FE head model generated from the ICBM 152 atlas is based on the same template as DTI, conductivity tensors derived from the ICBM DTI-81 atlas are directly mapped to the FE head model without geometrical adaption.

### Model Validation

**FIGURE 2:**
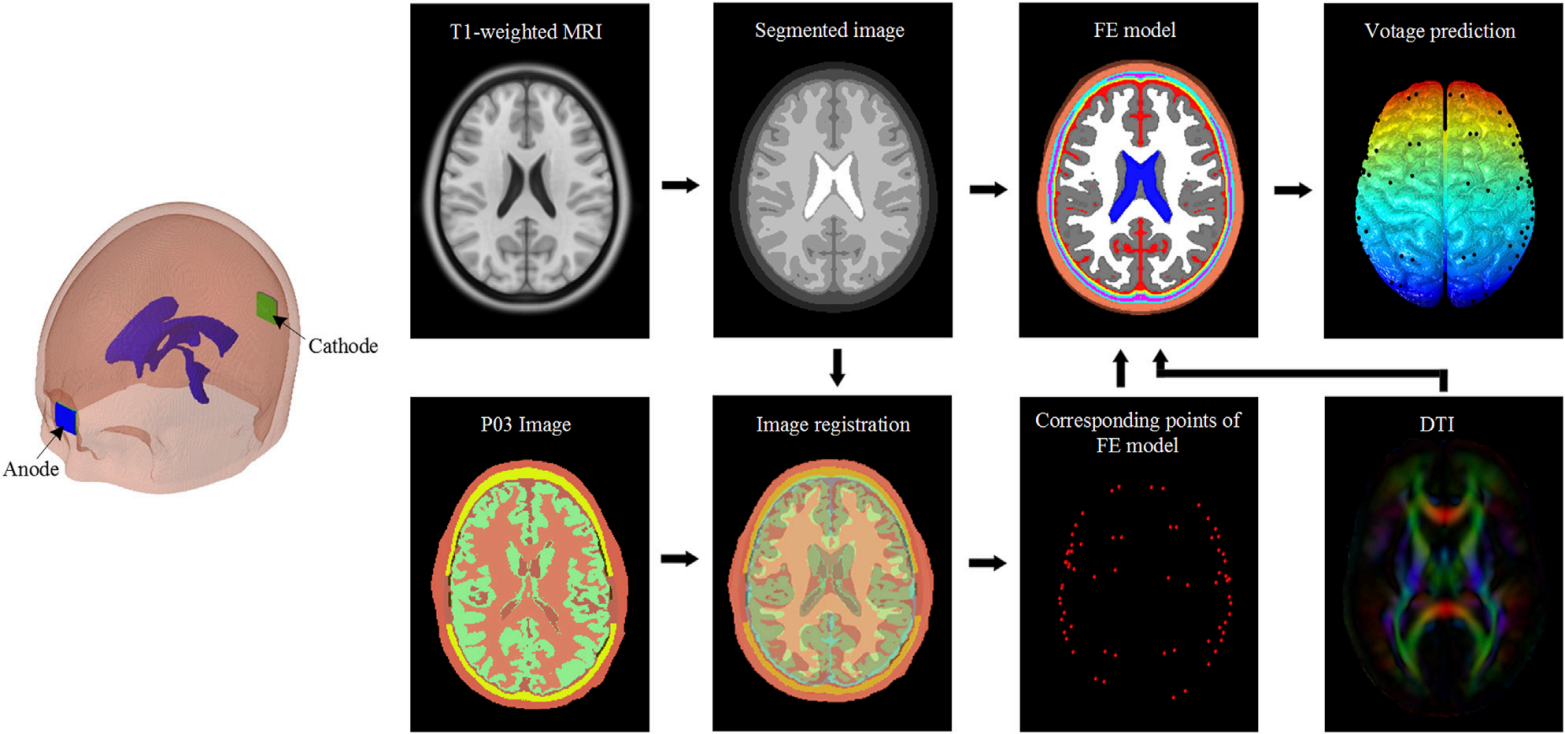
Model validation workflow. Patient images obtained from *in vivo* experiments (Huang et al., 2017) are affinely registered to the segmented images corresponding to the FE model used in the study. The obtained affine transformation matrix is then used to transform the recording electrodes coordinates of the patient to corresponding points of the FE head model. The diffusion tensor is used to derive the WM anisotropic conductivity. The FE head model and anisotropic conductivity tensor are then used to analyze the voltage distribution. The predicted values of voltage and calculated electric field are compared with *in vivo* experiments data reported by Huang et al. (2017).

The performance of the FE model is evaluated by comparing model-predicted voltage and electric field with *in vivo* experimental data in a patient (P03 in the original study) reported by Huang et al. (2017). The workflow of model validation is illustrated in Figure 2. Briefly, an anode pad (2 × 2 cm) is placed on the mid-forehead, and a cathode pad in the same size is placed at the occiput. Further, an inward current of 1 mA is applied to the anode while the cathode is set in contact with the ground. Both the locations of electrodes and applied current of 1 mA are the same as in experiments (Huang et al., 2017).

The predicted values at the corresponding points are extracted according to the coordinates of implanted electrodes *via* affine registration as shown in Figure 2. The electric field is recalculated by dividing the voltage difference of two adjacent electrodes by inter-electrodes distance. Pearson’s correlation coefficient (r) and Normalized root-mean-square error are calculated between model prediction and the experimental measurement. Model predictions from all three anisotropic models and the isotropic model are evaluated.

## Results

The influence of anisotropic algorithms on validation performance is presented first to show the effect of anisotropic WM on model prediction accuracy of voltage and electric field. Next, the electric field distribution of the three anisotropic WM is shown since EOF is proportional to the electric field magnitude according to Eq. 1. Finally, EOF is analyzed, which is related to the efficiency of the electroosmosis based approach for cerebral edema treatment.

### Model Validation Performance

**FIGURE 3:**
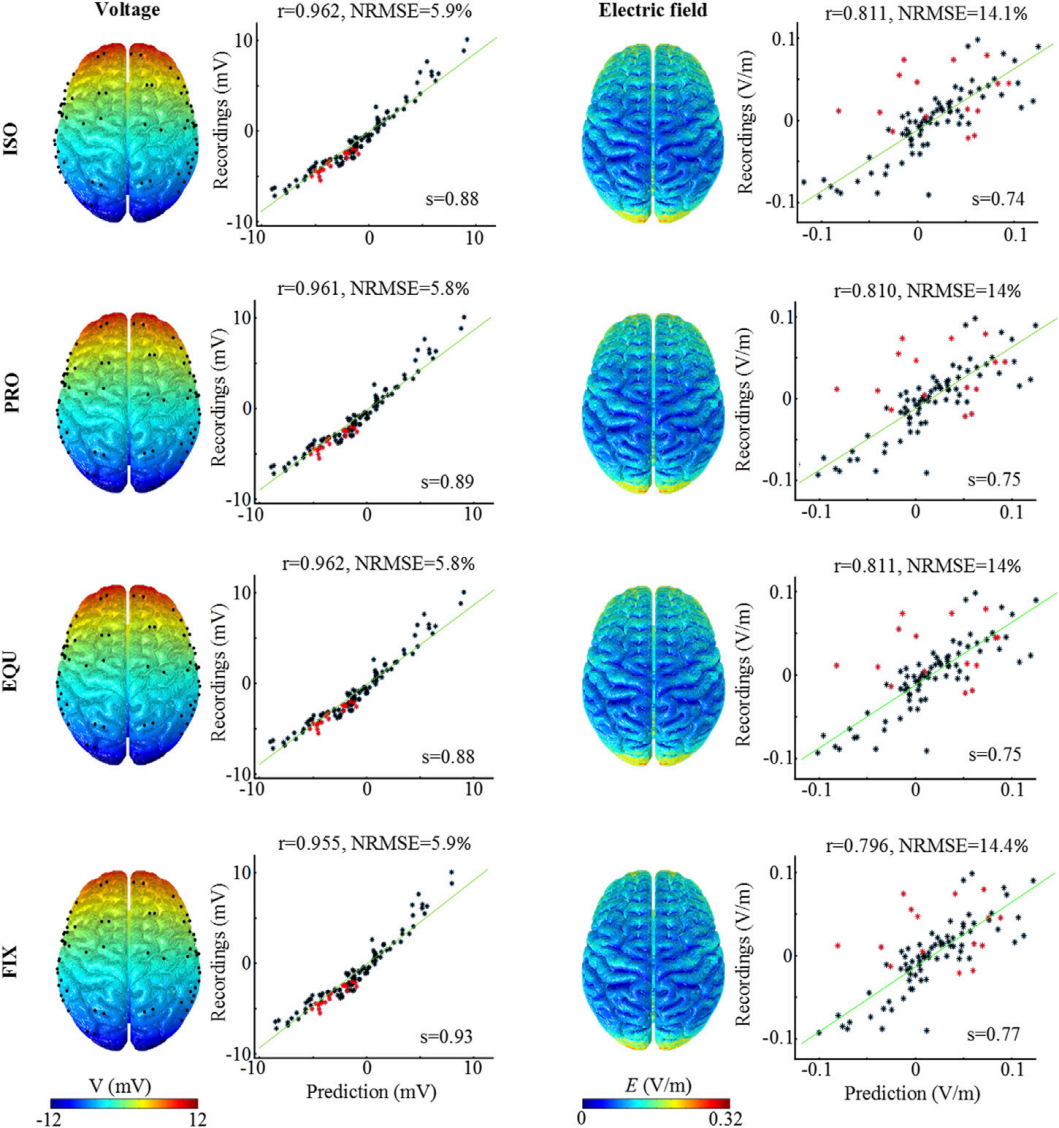
Measured and predicted voltage and electric field distribution. The first and third columns illustrate the predicted voltage and electric field distribution. The second and fourth columns show the correlation and difference analysis between measured and predicted values. The green line represents the fitted line, and s denotes the slope of the fitted line. r is Pearson’s correlation coefficient, while NRMSE represents the Normalized root-mean-square error (normalization is against the difference between the maximum and the minimum of the measured values, NRMSE in percent).

The measured voltage and electric field of the patient are compared to the predicted values for the three anisotropic models, as well as the isotropic model (Figure 3). The distribution of the predicted voltage and electric field shows no observable variation across the cortical surface by incorporating anisotropic WM. The correlation coefficient for voltage is 0.962 for ISO, 0.961 for PRO, 0.962 for EQU, and 0.955 for FIX, respectively, indicating that the predicted values are highly correlated with the measured voltages. A similar trend is also obtained for the electric field in which the FIX has the lowest correlation coefficient. Moreover, the results demonstrate that the FE models reasonably predict the voltage and electric field magnitudes as all slopes (s) of the fitted lines are higher than 0.73. For the differences (i.e., NRMSE) between predicted and measured values, the results show that the PRO and EQU have a better fit to the measured values compared to the ISO and FIX. The results indicate that all algorithms allow the FE head model to reasonably predict the distribution and magnitude of the electric field, especially the PRO and EQU have superior performance.

### Electric Field Distribution

**FIGURE 4:**
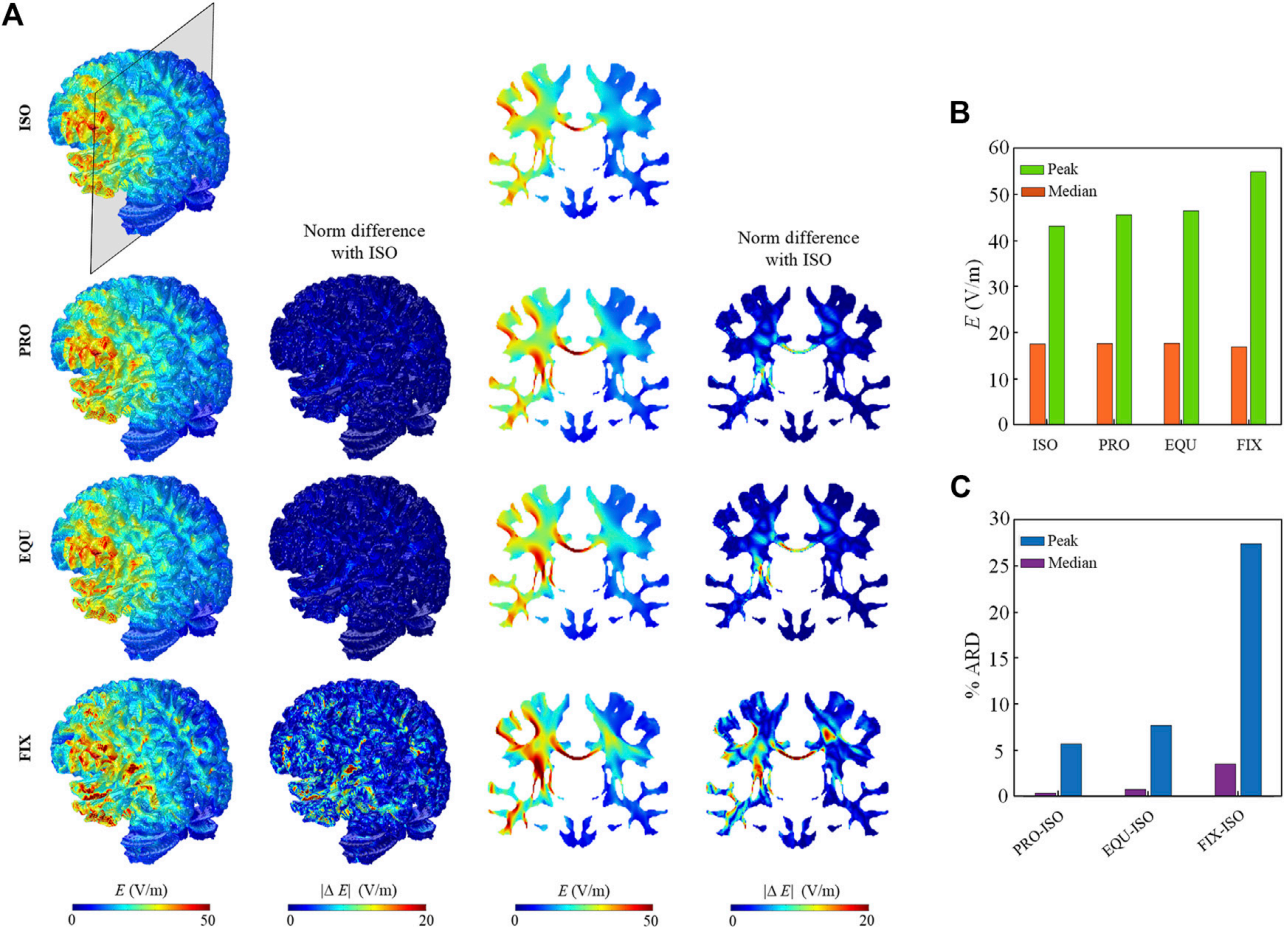
**(A)** Electric field distribution across the WM in the isotropic (ISO) and three anisotropic models (PRO, EQU, FIX). The first and third columns show the distribution of the electric field on the WM surface and coronal planes. The second and fourth columns show the norm of difference in the anisotropic models relative to the isotropic model. **(B)** Peak and median values of electric field for WM in the left hemisphere for four models. **(C)** The percent ARD between isotropic and each anisotropic model for peak and median values.

The predicted electric field distribution in the WM and the norm of the difference between anisotropic and isotropic models are evaluated (Figure 4). Electric field distribution at WM surface is similar among ISO, PRO, and EQU models, while FIX exhibits a higher magnitude underneath the anode (Figure 4A). Further, a higher electric field is observed in the corpus callosum and internal capsule in all three anisotropic models than ISO due to low conductivity across the WM fibers. Since the activated region is mainly located between anode and cathode at the left hemisphere, the peak (calculated as the 99^th^ percentile) and median (calculated as the 50^th^ percentile) of the electric field magnitude for WM in the left hemisphere (Figure 4B) are calculated. The absolute relative difference (ARD) (Figure 4C) is estimated by dividing the value difference of the isotropic and each anisotropic model by the value of the isotropic model. The results indicate the peak values in anisotropic models are noticeably affected due to incorporating anisotropic WM while the anisotropic conductivity exhibits a slight effect on median values.

### Electroosmotic Flow Distribution

**FIGURE 5:**
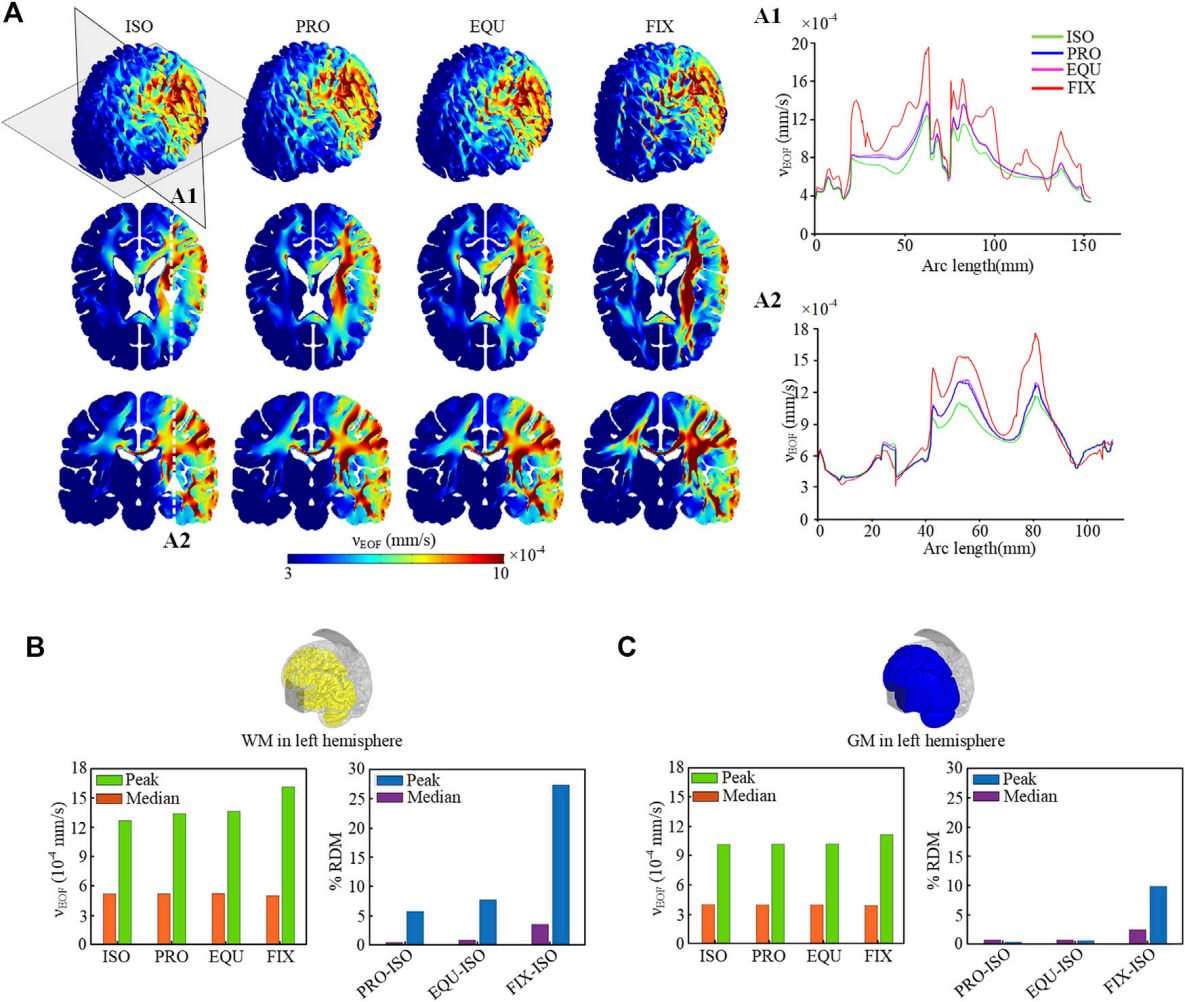
**(A)** Distribution of EOF velocity across the brain and on the axial and coronal planes. The EOF velocity along the crosslines in two brain areas on the axial **(A1)** and coronal **(A2)** planes in evaluated quantitatively. **(B)** Peak and median values of EOF for WM in the left hemisphere for four models, and the percent ARD between isotropic and each anisotropic model for peak and median values. **(C)** Peak and median values of EOF for GM in the left hemisphere and the percent ARD.

High EOF velocity is only observed on the activated regions under and between anode and cathode, whereas the magnitude of EOF velocity is relatively low on the opposite hemisphere (Figure 5A). The higher values of EOF velocity in WM are attributed to the lower average conductivity compared with GM. As shown in coronal planes, the EOF velocity induced in the corpus callosum and surrounding internal capsule in the anisotropic models is faster than that in the ISO due to the anisotropic conductivity, especially in FIX with the largest anisotropic ratio. The quantitative results along two crosslines (Figures 5A1,A2) demonstrate that three anisotropic models show noticeable differences in EOF velocity compared to the isotropic model, of which the FIX model shows the highest degree of variation. Similarly as electric field, the activated region for EOF is mainly located at the left hemisphere, the peak (percentile) and median (calculated as the 50^th^ percentile) of EOF velocicalculated as the 99^th^ ty magnitude in the left WM (Figure 5B) and GM (Figure 5C) are calculated, respectively. The results show that anisotropic models exhibit noticeable differences in peak velocity compared to the isotropic model, indicating the anisotropic effect on EOF magnitude is mainly located in the WM and limited in the GM. In addition, given that an essential condition to induce EOF inside the brain is the narrow channels with charged walls as in the extracellular space, the electric field mainly induces EOF in the brain parenchyma and does not cause EOF in the CSF system nor in the ventricles.

### Electroosmotic Flow Direction

**FIGURE 6:**
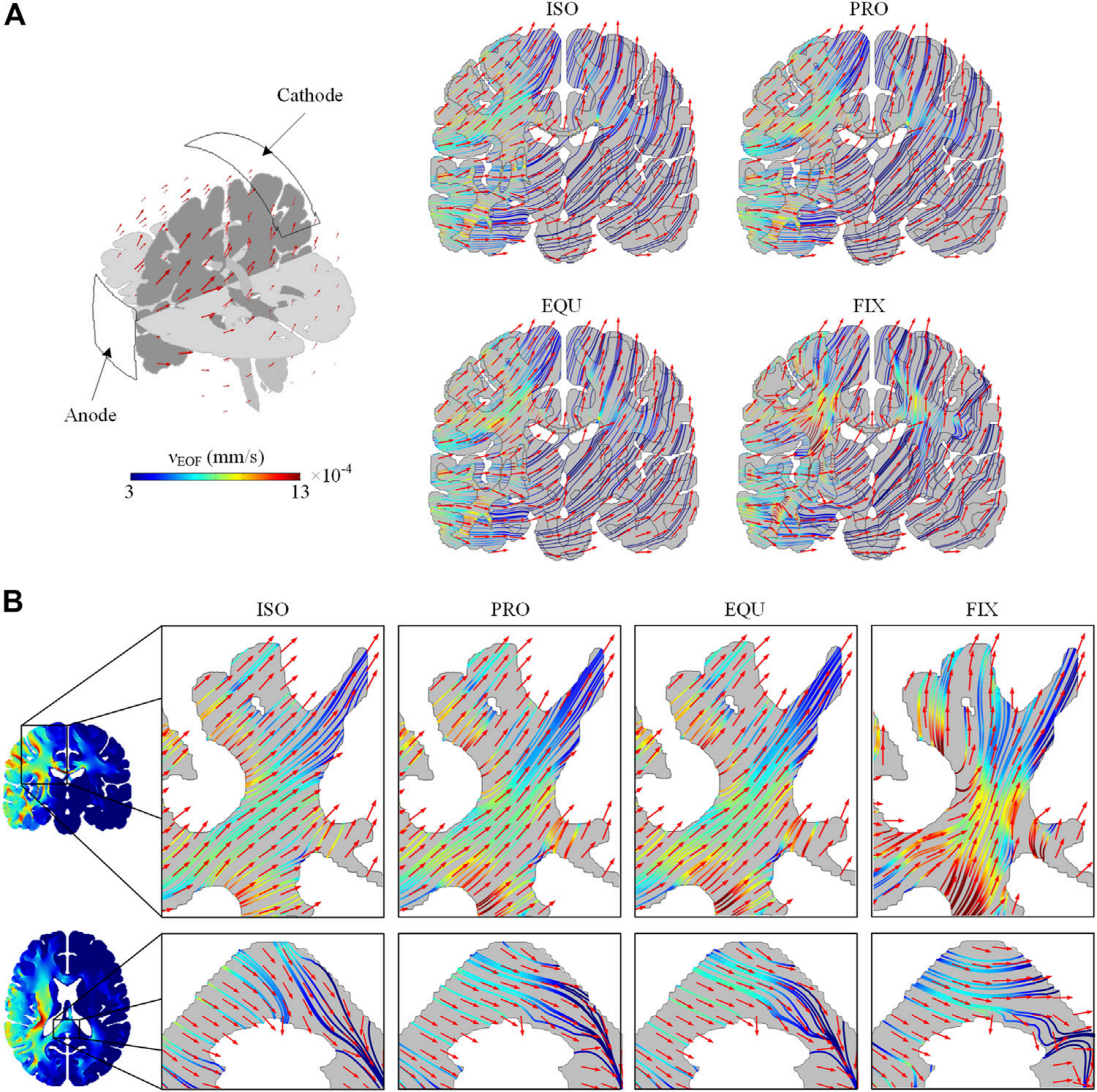
**(A)** The pathways and direction of EOF across the brain in ISO, PRO, EQU, and FIX models on a coronal plane. **(B)** The pathways and direction of EOF in corona radiata on a coronal plane and the splenium of the corpus callosum on an axial plane. The color of EOF pathways denotes the EOF magnitude. The red arrows indicate the direction of induced EOF.

The induced EOF pathways (colored lines) and direction (red arrows) across the brain are plotted (Figure 6). As the induced EOF is mainly directed by the electrode placement and boundary condition, the EOF flows from regions close to the anode to SAS underneath the cathode (Figure 6A). Compared to the isotropic model, the EOF pathways in PRO and EQU exhibit a slight deviation, whereas the FIX gives a higher degree of deviation due to the larger anisotropic ratio. Especially in the corona radiata and splenium of the corpus callosum, the EOF shows preferred direction parallel to the fiber orientation when anisotropic conductivity is incorporated in PRO and EQU (Figure 6B). Moreover, the EOF direction in the FIX model is substantially parallel to the fiber tracts.

## Discussion

We present a novel approach for mitigating cerebral edema by inducing bulk fluid transport (i.e., EOF) within the brain parenchyma by electric field utilizing brain tissue’s electroosmotic property, and investigate the effect of WM anisotropic conductivity on the induced EOF. The WM anisotropy shows a significant influence on the distribution and direction of the induced EOF across the brain by affecting the current flow pathways and electric field distribution. The direction of the induced EOF in the anisotropic models deviates from its isotropically defined pathways and tends to move along the principal fiber direction due to the existence of anisotropic WM. Thus, WM anisotropy is an essential factor for a reliable prediction of EOF for cerebral edema treatment. The results show the promise of the proposed electroosmosis based approach to be developed as a new therapy for edema treatment as evaluated with enhanced head models incorporating WM anisotropy.

When direct current is applied to the edema regions, the mobile cations pull the surrounding fluid involving charged and neutral particles across the extracellular space, which can reduce the accumulation of ions and water simultaneously. Since the cathode is placed close to the SAS and the CSF velocity around the cortical surface is significantly higher than the induced EOF, the excess fluid driven to the SAS will be absorbed into SSS together with CSF, resulting in the drainage of excess fluid and the decrease of ICP. Moreover, a significant stimulation-polarity-specific fluid and solute movement is induced across endothelial cell monolayers under applied direct current stimulation, indicating the excess fluid can be driven across the blood-brain-barrier (BBB) to achieve a decrease of water content in brain parenchyma (Cancel et al., 2018). In our previous study, a localized edema with an excess fluid volume of 4.8 ml was assumed underneath the anode (von Holst et al., 2012; Wang et al., 2020), and the proposed approach allows driving excess fluid out of the edema regions at a rate of 2.38 ml/h based on our preliminary studies. Moreover, the proposed approach might be designed towards a complement to hyperosmotic therapy, in which the excess fluid is driven out of the edema region and then absorbed into the vascular system by hyperosmotic therapy. In addition, the induced EOF in other regions without electrode placement exhibits relatively lower velocity and the EOF effect mainly exists in the region between the anode and cathode, suggesting that undesired outcomes in other regions can be avoided.

WM mainly consists of bundles of myelinated nerve cell axons, in which myelin sheath forms an impermeable boundary obstructing the diffusion of particles (Stanisz, 2003; Wu et al., 2018). Therefore, the oriented distribution of nerve fibers constrains the diffusion process of water molecules. Given the similarity between the transportation processes of charged particles and water molecules (Lee et al., 2012; Wu et al., 2018), the WM is believed to achieve anisotropic conductivity and facilitate current flow more parallel to the principal fiber direction than perpendicular. Especially in regions with a high anisotropic ratio (e.g., corpus callosum and corona radiata), the current flow is more aligned to the principal fiber direction. As the induced EOF is based on the movement of cations by viscous drag, the EOF pathways are parallel to the current flow under an external electric field. Thus, the EOF tends to move along the principal fiber direction due to the existence of WM anisotropy. According to the Helmholtz–Smoluchowski approximation, the EOF velocity value is proportional to the electric field magnitude. The anisotropic conductivity has a significant effect on the electric field distribution in the interior WM, resulting in the variation of the EOF distribution. A relatively high electric field is induced in the corpus callosum due to lower conductivity perpendicular to the fiber orientation, resulting in high predicted EOF velocity (Figure 5), which overestimates the EOF velocity to some extent due to the lack of fiber tract resistance to EOF in the simulation. Besides, a significant effect of WM anisotropy on the electric field and EOF velocity distribution is mainly found in the WM and to a lesser extent in the GM. In this study, the results of model validation show a limited effect of WM anisotropy on the cortical surface (Figure 3), in line with previous studies (Wagner et al., 2013; Huang et al., 2017) in which the FE head model with isotropic conductivity is effective in predicting the distribution of the electric field on the cortical surface. However, when we focus on the electric field or EOF inside the brain, especially in the WM, it is imperative to incorporate the WM anisotropy to achieve a more accurate prediction according to our findings in the current study.

The three representative algorithms (i.e., PRO, EQU and FIX) widely used in tDCS (Shahid et al., 2013; Huang et al., 2017), electroconvulsive therapy (Lee et al., 2012) and electroencephalography source location (Hallez et al., 2008; Lee and Kim, 2012) are employed to generate anisotropic conductivity of the WM. Both PRO and EQU algorithms are based on the heterogeneous and anisotropy orientation information from DTI directly, in which the eigenvalues and eigenvectors are calculated at a voxel level based on the relationship between diffusion tensor and conductivity tensor. Thus, both algorithms reflect the variation of anisotropic ratio among different regions of the WM and result in a similar and superior prediction of electric field and EOF. Given the eigenvalues obtained from the PRO algorithm are constrained by the volume of isotropic conductivity tensor (Eq. 6), PRO algorithm poses an advantage of retaining the geometric mean of the eigenvalues compared to the EQU algorithm. Although FIX algorithm is also widely used to calculate the anisotropic conductivity of the WM due to easy processing and retaining the original orientation of the diffusion tensor (Hallez et al., 2008; Lee et al., 2012; Shahid et al., 2013; Huang et al., 2017), it appears more suitable for analyzing the electric field on the cortical surface, in line with our findings in the model validation in which the FIX model also shows a reasonable prediction. However, as FIX algorithm ignores heterogeneity throughout the WM in which the anisotropic ratio varies among different regions (Hallez et al., 2008), the results from FIX algorithm in the current study exhibit significant variation compared to other anisotropic or isotropic models.

For the safety criteria, the minimum induced current density to cause brain damage in rats is predicted as 12 A/m^2^ based on the rat experiments (Liebetanz et al., 2009; Bikson et al., 2016), which is higher in comparison to the induced peak current density of around 10.51 A/m^2^ for ISO, 10.51 A/m^2^ for PRO, 10.53 A/m^2^ for EQU, and 10.33 A/m^2^ for FIX in our study. On the other hand, Liebetanz et al. (2009) studied the rat brain damage under the cathodal tDCS and found that the brain damage was caused with an applied direct current of 500 μA for 10 min, whereas no brain lesion is observed under the same current strength for 3.33 min. Therefore, intermittent treatment is expected to be another therapy choice with respect to the therapeutic schedule with low direct current in an uninterrupted duration. Moreover, the electric field necessary to electroporate a cell is up to 6.7 × 10^3^ V/m (Nolkrantz et al., 2001). Since the EOF magnitude exhibits a linear correlation with the electric field, the applied direct voltage can be adjusted based on the safety criteria. To confirm the safety of applied voltage dose for certain electrode configurations, animal experiments based on this design should be implemented in future work.

A smooth-voxel approach (Boyd and Müller, 2006) is used to generate hexahedral elements in the head model in this study. This approach is efficient for generating FE head models by converting 1-mm segmented voxels directly to a FE element after smoothing, which produces head models with anatomical details. The 1-mm element size adopted in this study also facilitates incorporating anisotropic conductivity of WM having the same resolution as DTI image, allowing eigenvalues and eigenvectors of the electrical conductivity calculated at each white matter voxel applied to corresponding hexahedral element directly. To further motivate the current choice of hexahedral mesh, a convergence study is carried out to study the sensitivity of the FE head model response to mesh discretization by increasing mesh resolution (Supplementary Material). The results demonstrate that FE model with hexahedral element at 1 mm resolution is converged which brings confidence to the model predictions.

Some limitations need to be mentioned for this study. As the purpose of this study is to investigate the effect of anisotropic WM on the magnitude and direction of EOF across the brain, a baseline FE head model under normal conditions is employed to compare the EOF variation between different models instead of explicitly simulating brain edema. Further, during the electroosmosis based treatment, brain deformation is expected to occur, which is not accounted for in the current study but is an important aspect to include in the future to further evaluate the treatment efficiency of the proposed approach. Moreover, the induced EOF in the brain will be further validated to confirm its prediction accuracy by data obtained from the future animal or clinical experiments.

In conclusion, this study provides an insight into the variation in the EOF distribution and direction across the brain due to the presence of anisotropic conductivity. In contrast to the isotropic model, anisotropic models exhibit a substantial effect of anisotropic conductivity on the EOF magnitude and direction, which are essential for the evaluation and design of the proposed electroosmotic edema treatment approach. These results also demonstrate that the PRO algorithm is suitable to incorporate directional conductivity for a more realistic prediction of the induced EOF for evaluating electroosmosis based treatment of cerebral edema.

## Data Availability

Publicly available datasets were analyzed in this study. This data can be found here: http://crcns.org/data-sets/methods/tes-1.
