# Supplementary material for: Influence of Anisotropic White Matter on Electroosmotic Flow Induced by Direct Current
Source: Front Bioeng Biotechnol. 2021 Aug 13;9:689020. doi: 10.3389/fbioe.2021.689020 (PMC8414365; doi:10.3389/fbioe.2021.689020)
Supplement: Supplementary file 1 [file DataSheet1.PDF]

Supplementary Material for

"Influence of anisotropic white matter on electroosmotic flow induced by direct current"

Teng Wang[1]*, Svein Kleiven[1], Xiaogai Li[1]

[1]Division of Neuronic Engineering, Department of Biomedical Engineering and Health Systems, KTH Royal Institute of Technology, Huddinge 141 52, Sweden

*Correspondence: tenwan@kth.se

Mesh discretization on simulation accuracy

A convergence study is carried out to study the sensitivity of the FE head model with isotropic conductivity response to mesh discretization by increasing mesh resolution. Four head models are constructed with varying element size based on the same segmented MR image. The numbers of hexahedral elements are 8119826 for the head model with mesh resolution of 0.75 mm, 3445410 for the head model with mesh resolution of 1 mm, 1021165 for the head model with mesh resolution of 1.5 mm, and 431164 for the head model with mesh resolution of 2 mm. The anode pad (2 × 2 cm) is placed on the mid-forehead while the cathode pad in the same size is located at the occiput. The identical direct current of 1 mA is applied to the anode, and the cathode is set in contact with the ground.

The electric field on the cortical surface and inside the GM (Fig. 1) is calculated, and the norm of the difference between the electric field in the three lower resolutions and the electric field in the highest resolution of 0.75 mm is considered as the errors ΔE. As shown in the left small panels, the finer element leads to a more accurate representation of anatomical features on the surface of gray matter. The anatomical details of the GM boundary in the gyri and sulci are better preserved at higher mesh resolution. The results show similar electric field distribution of GM, indicating that the head models with four different resolutions all give a good overview of the electric field distribution. However, the magnitude of the electric field is significantly affected as the mesh resolution increases, especially in the regions underneath the electrodes. The absolute errors of the electric field indicate that the electric field in head mode with 1 mm resolution is closest to that with 0.75 mm resolution, followed by the head model with a 1.5 mm resolution, while the head model with 2 mm resolution is the worst. Since the different mesh resolution also causes the change of anatomical features, the reasons to affect numerical accuracy in this study are not only mesh resolution, but also the anatomical fidelity.

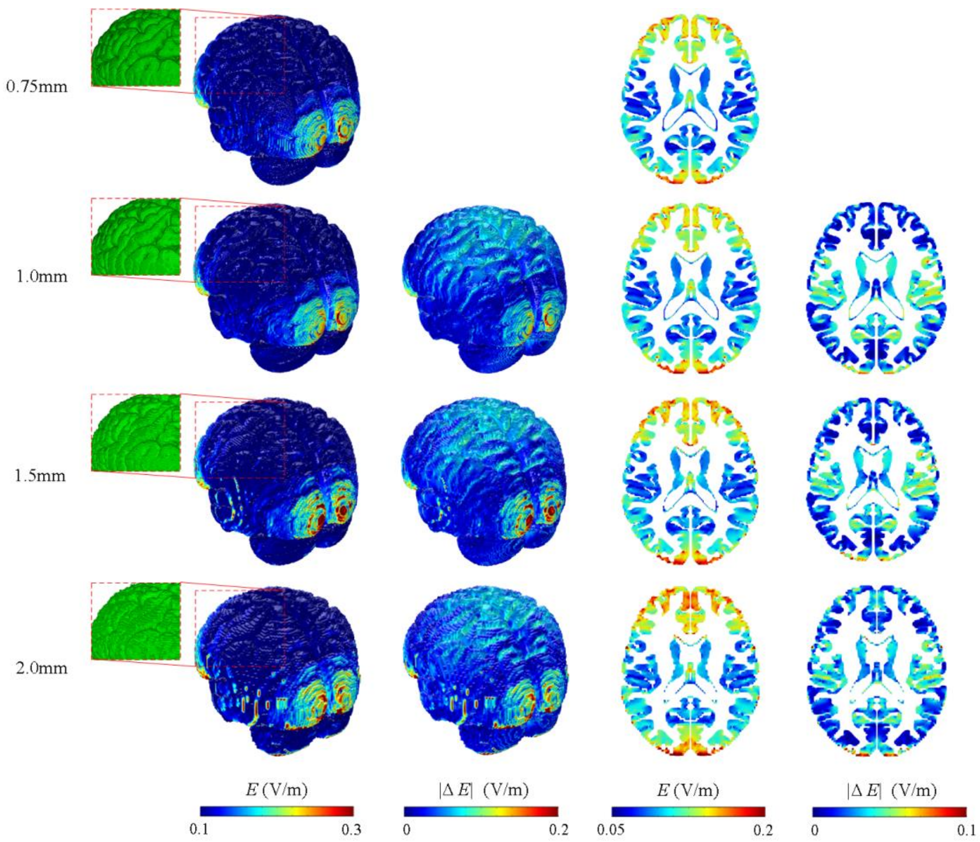

Figure 1. Distribution of electric field and absolute errors relative to the highest resolution model on the cortical surface and inside the GM.

The results (Fig. 2) show that both the peak (i.e., the 99th percentile) and median (i.e., the 50th percentile) values of the electric field are sensitive to the mesh discretization. The errors are calculated with respect to the electric field calculated using the 0.75 mm element resolution. The peak and median values with mesh resolution at 1 mm and 1.5 mm show a good agreement with that at 0.75 mm resolution, which is considered "converged". However, compared to the anatomical features of gray matter with a 1.5 mm resolution, the head model with a 1 mm resolution represents the brain anatomical features better. Thus, the converged model should also have a good anatomical fidelity in order to have a better understanding of different parts of the brain.

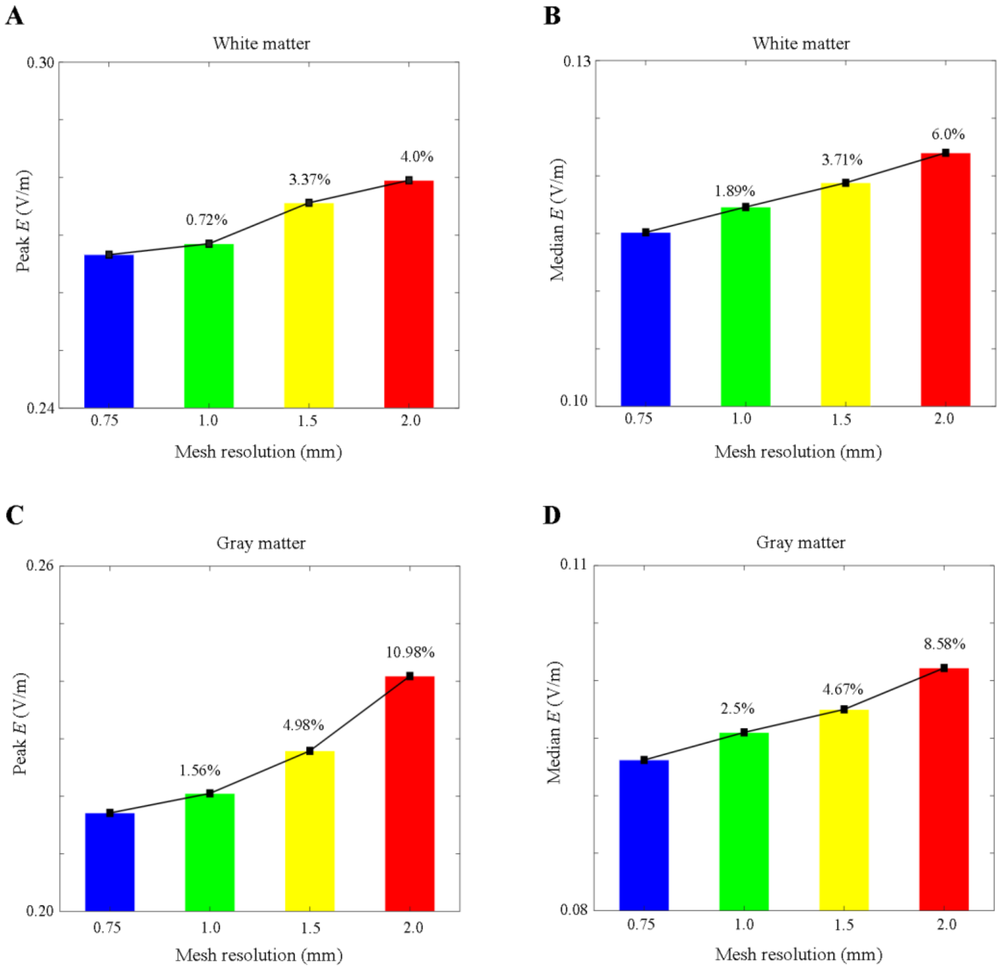

Figure 2. Bar charts show the peak and median values of electric field of head models with four different resolutions. (A) Peak values of electric field in WM. (B) Median values of electric field in WM. (C) Peak values of electric field in GM. (D) Median values of electric field in GM.
